# Benchmarking veterinary librarians’ participation in systematic reviews and scoping reviews

**DOI:** 10.5195/jmla.2019.710

**Published:** 2019-10-01

**Authors:** Lorraine Toews

**Affiliations:** Librarian, Veterinary Medicine and Bachelor of Health Sciences, Health Sciences Library, 3330 Hospital Drive Northwest, University of Calgary, Calgary, AB T2N 4N1, Canada, and Adjunct Associate Librarian, Department of Ecosystem and Public Health, Faculty of Veterinary Medicine, 3280 Hospital Drive Northwest, University of Calgary, Calgary, AB T2N 4Z6, Canada, ltoews@ucalgary.ca

## Abstract

**Objectives:**

The objectives of this study were to benchmark roles that veterinary librarians at universities and colleges play in systematic reviews (SRs) and scoping reviews that are conducted by faculty and students at their institutions, to benchmark the level of training that veterinary librarians have in conducting SRs, to identify barriers to their participation in SRs, and to identify other types of literature reviews that veterinary librarians participate in.

**Methods:**

Sixty veterinary librarians in universities and colleges in Canada, the United States, England, Scotland, Ireland, Australia, and New Zealand were surveyed online about their roles and training in conducting SRs, barriers to participation in SRs, and participation in other types of literature reviews.

**Results:**

Veterinary librarians’ highest participation was at an advising level in traditional librarian roles as question formulator, database selector, search strategy developer, and reference manager. Most respondents reported pretty good to extensive training in traditional roles and no or some training in less traditional roles. Sixty percent of respondents received few or no requests to participate in SRs, and only half of respondents had participated in SRs as a review team member. Sixty percent of respondents stated that their libraries had no policies regarding librarian roles and participation in SRs.

**Conclusions:**

The surveyed veterinary librarians participated in SRs to a lesser degree than human health sciences librarians, experienced low demand from veterinary faculty and students to participate in SRs, and participated as review team members at significantly lower rates than human health sciences librarians. The main barriers to participation in SRs were lack of library policies, insufficient training, and lack of time.

## INTRODUCTION

Well-conducted systematic reviews (SRs) and scoping reviews are types of research knowledge syntheses that are important resources for supporting evidence-based veterinary practice and identifying gaps in the veterinary literature where additional research is needed [1]. Knowledge synthesis reviews use reproducible and transparent methods to contextualize and integrate individual research studies within the larger body of research knowledge on a topic [2]. Current best practice guidelines for conducting SRs recommend including librarians on review teams [3, 4], and some research studies indicate that including a librarian on SR teams to plan, conduct, and report the literature search are associated with better quality and more reproducible literature searches [5–7]. Despite this, anecdotal evidence suggests that veterinary librarians working at universities and veterinary colleges do not participate in SRs and scoping reviews to nearly the same degree as academic librarians in the human health sciences. This study sought to understand if this was, in fact, the case and to explore reasons for this difference.

Published studies have benchmarked the roles that health sciences librarians play in conducting SRs and scoping reviews [7–11], but to date no published studies have focused specifically on veterinary librarians. In 2003, Beverley et al. identified ten roles for librarians in the SR process, including project leader, project manager, literature searcher, reference manager, document supplier, critical appraiser, data extractor, data synthesizer, report writer, and disseminator [8]. Murphy and Boden surveyed Canadian academic health sciences librarians about their participation in SRs, expanding beyond the literature searcher role that Beverley identified to include question formulator, database selector, and search strategy developer, and adding three levels of participation: adviser, formal teacher, and review team member. Murphy and Boden found that over half of respondents participated in the traditional librarian functions of searcher and reference manager, while less than half participated in any of the nontraditional roles. Lack of time and insufficient training were the most frequently reported barriers to participation in SRs [9]. In a 2016 literature review that mapped out potential functions for librarians in scoping reviews, Morris et al. recommended that librarians could best contribute in the same roles that Beverley et al. and Murphy and Boden identified: question formulator, search strategy developer, reference manager, and report writer [10].

The objectives of this study were: (1) to benchmark the roles that veterinary librarians working at universities and veterinary colleges play in SRs and scoping reviews that faculty members and students at their institution conduct, (2) to benchmark the level of training that veterinary librarians have received in conducting SRs and scoping reviews, (3) to identify barriers to their participation in SRs and scoping reviews, and (4) to identify other types of literature reviews that veterinary librarians participate in.

## METHODS

This study received ethics approval from the University of Calgary Conjoint Faculties Research Ethics Board on April 23, 2018. An online survey (supplemental Appendix A), generated using Springshare’s LibWizard software [12], was distributed on April 24, 2018, via individual emails sent to sixty veterinary librarians working in universities and veterinary colleges in Canada, the United States, England and Scotland in the United Kingdom, Ireland, Australia, and New Zealand. These countries were selected because they are primarily English-speaking and because most universities and colleges with veterinary programs in these countries have librarians on staff, whose duties include providing support for veterinary teaching, learning, and research and so would be expected to participate in SRs.

A convenience sample of librarians was identified using the online Veterinary Medical and Related Libraries International Directory, which is a noncommercial directory compiled by volunteers from the Veterinary Medical Libraries Section of the Medical Library Association [13]. Veterinary librarians were identified by job titles listed in the directory or on the librarian’s institutional employer website. Librarians whose job title was ambiguous or could not be determined; agriculture and life sciences librarians; librarians in zoo, government, or veterinary clinic libraries; and paraprofessional library support staff were excluded from the study sample. Within the limitations imposed by absent or ambiguous job titles, the final sample of sixty librarians who were contacted represented most of the veterinary librarians listed in the directory for each of the designated countries and represented a total of forty-nine unique institutions.

The survey contained six questions including Likert, matrix, single answer multiple choice, multiple answer multiple choice, and open-ended short answer formats. Questions about various roles that librarians played in SRs and scoping reviews were derived from Beverley et al. [8]. These roles included project leader, project manager, research question formulator, database selector, search strategy developer, reference manager, article selector, data extractor, article appraiser, data synthesizer, and report writer. Respondents were asked to indicate one of three levels of participation in SRs for each role, adapted from Murphy and Boden [9]: providing expert advice to students and faculty, teaching formal classes to students and faculty, or participating as a member of a review team. The latter is usually the most intensive and can include receiving formal credit as a coauthor of the review [14].

The survey also asked veterinary librarians to rate their levels of training in SR methods, identify barriers to their participation in SRs, indicate whether their libraries had policies or guidelines regarding librarian participation in SRs, and report on their participation in other types of literature reviews. No personal identifying information such as names, email addresses, phone numbers, country of employment, or Internet protocol (IP) addresses were collected in the survey.

Survey results were exported into Microsoft Excel to facilitate data analysis (supplemental Appendix B). Text-based data were anonymized by stripping out names of persons, places, and organizations that might serve to identify individuals or specific organizations. All survey responses were included and analyzed, even for surveys that were only partially answered. Descriptive statistics were calculated for questions two to five, and text responses in question six were analyzed for themes.

## RESULTS

Twenty-five veterinary librarians completed the survey for a response rate of 42%.

### Roles

**Figure 1:**
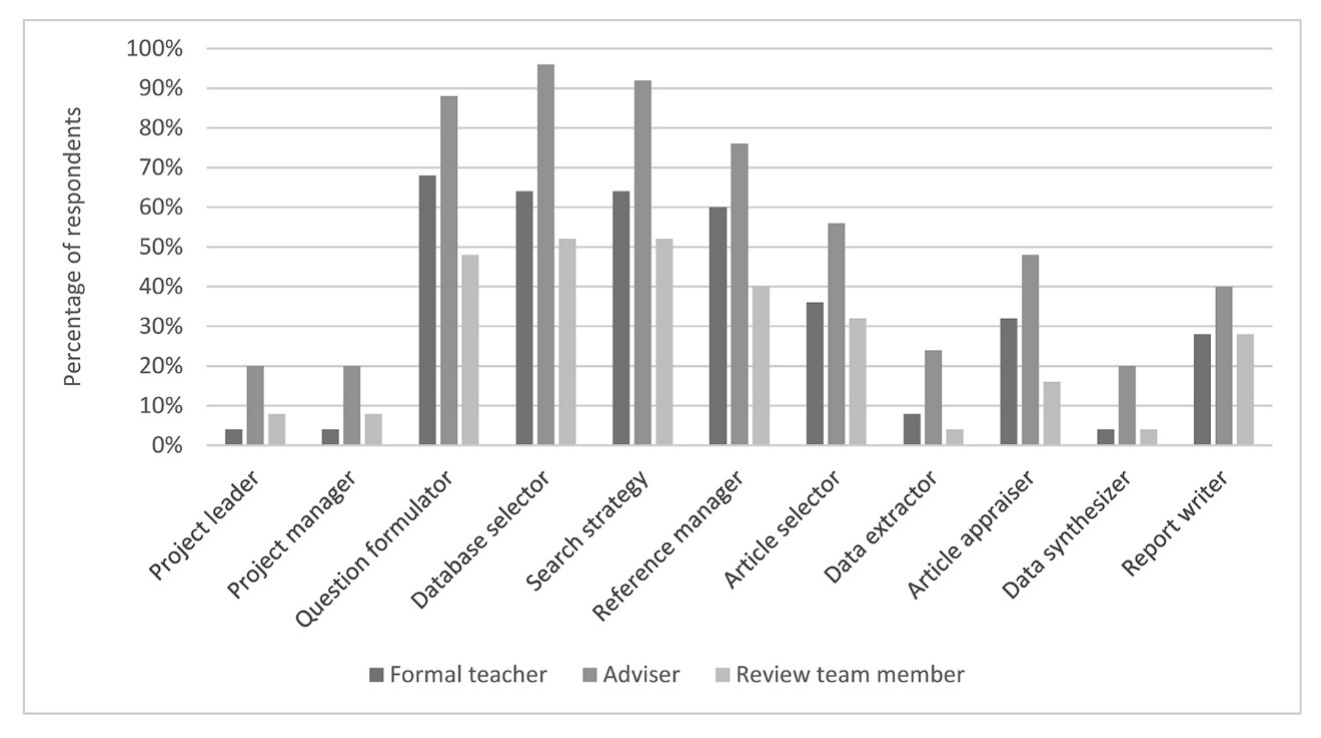
Veterinary librarian roles in systematic and scoping reviews

All respondents (n=25, 100%) answered the survey question regarding their participation in various SR and scoping review roles. Each cluster of bars in Figure 1 represents 1 of the 11 roles that veterinary librarians played in conducting SRs [8]. Within each cluster, the 3 bars represent 3 levels of participation, as identified by Murphy and Boden [9]: adviser, teacher of formal classes to students and faculty members, and member of a review team. Veterinary librarians reported the highest levels of participation in the traditional librarian roles of question formulator, database selector, search strategy developer, and reference manager, and much lower levels of participation in nontraditional roles.

With regard to level of participation, veterinary librarians reported advising as highest in all 11 roles, followed by formal teaching and participating as a review team member. Respondents reported that they acted as an adviser in their roles as question formulator (n=22), database selector (n=24), search strategy formulator (n=23), and reference manager (n=19). With respect to formal teaching, respondents indicated that they had focused on their expertise as question formulators (n=17), database selectors (n=16), search strategy formulators (n=16), and reference managers (n=15). Only half of veterinary librarians reported that they had participated as a review team member during the past 3 years as question formulator (n=12), database selector (n=13), search strategy developer (n=13), or reference manager (n=10).

### Barriers

**Figure 2:**
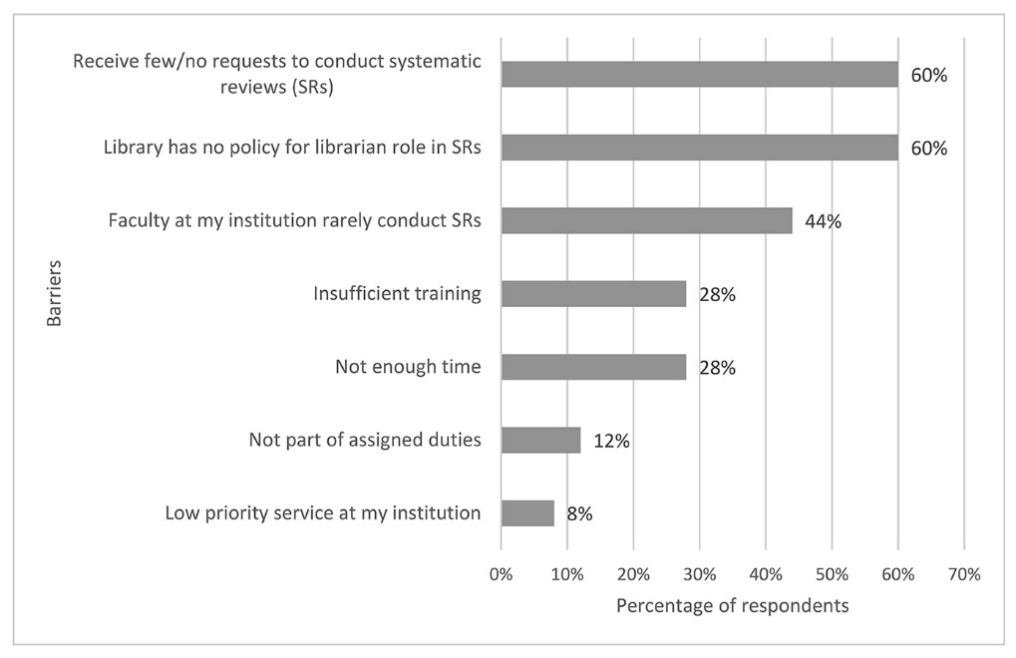
Barriers to veterinary librarian participation in systematic and scoping reviews

Of the 25 survey respondents, 24 (96%) replied to the question on barriers that limited their ability to participate in SRs and scoping reviews. As shown in Figure 2, the most frequently reported barrier was a lack of requests (n=15) from faculty and students for veterinary librarians to participate in conducting reviews. A second most common barrier was the lack of library policies and guidelines regarding librarian roles and participation in SRs, with nearly two-thirds (n=15, 60%) of respondents indicating that their library had no policy. The third most commonly reported barrier was the observation that veterinary faculty at the respondents’ institutions rarely conducted SRs (n=11). While other barriers were less frequently referenced, insufficient training (n=7) and lack of time (n=7) were identified by more than one-quarter of respondents.

### Training

Of the 25 survey respondents, 23 (92%) fully answered the question about their levels of training in various roles in conducting SRs. One (4%) librarian answered all parts of the training question except for the project management role, and 1 (4%) librarian answered only the parts of the training question dealing with the roles of selecting databases and developing and conducting the search strategy. Details are in supplementary Appendix B in the associated data.

**Figure 3:**
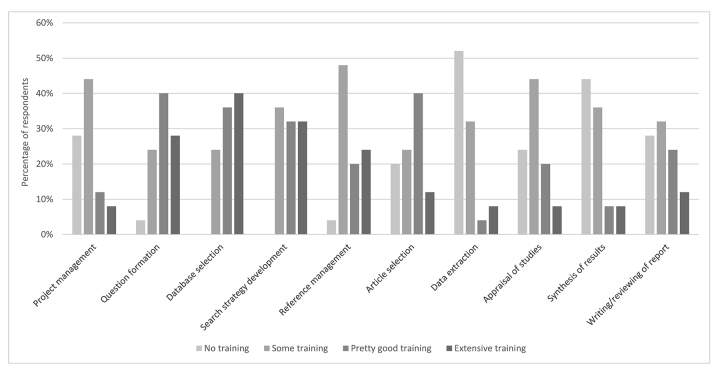
Training levels of veterinary librarians

Figure 3 details veterinary librarians’ ratings of their levels of training in conducting SRs. In traditional librarian roles, respondents rated their levels of training as extensive in database selection (n=10), search strategy development (n=8), question formation (n=7), and reference management (n=6). In these same roles, respondents rated their levels of training as pretty good in question formation (n=10), database selection (n=9), search strategy development (n=8), and reference management (n=5). By contrast, respondents stated that they had some level of training in SR roles that are less traditional for librarians: appraisal of studies (n=11), synthesis of results (n=9), data extraction (n=8), writing of review reports (n=8), and article selection (n=6). Finally, respondents rated their level of training as none for data extraction (n=13), synthesis of results (n=11), writing review reports (n=7), appraisal of studies (n=6), and article selection (n=5).

### Other types of literature reviews

Less than half (n=11, 44%) of the 25 respondents answered the final survey question regarding their participation in other types of literature reviews. Responses were diverse, with the greatest number of veterinary librarians involved in narrative reviews (n=3, 12%) and dissertations or theses (n=3, 12%), followed by case reports (n=2, 8%). Individual librarians also reported their involvement in literature reviews for faculty or student book chapters, conference papers, course assignments, rapid reviews, and patent proof of concept.

## DISCUSSION

### Roles

In this survey, veterinary librarians were similar to their counterparts in the human health sciences in reporting the highest levels of participation in the traditional librarian roles of question formulator, database selector, search strategy developer, and reference manager and much lower levels of participation in nontraditional roles [9]. Veterinary librarians were also similar to human health sciences librarians in participating most frequently in an adviser function [9]. However, as a striking difference, veterinary librarians participated as review team members at much lower rates than their human health sciences counterparts, with only half of veterinary librarians having participated as review team members in the four traditional librarian roles. In contrast, more than three-quarters of human health sciences librarians surveyed by Murphy and Boden [9] participated as review team members in these same four traditional roles.

### Barriers

With regard to barriers to participation in SRs, one of the most significant findings of this study was the lack of demand from faculty and students for veterinary librarians to participate in SRs, with almost two-thirds of respondents indicating they had received few or no requests and slightly less than half of respondents reporting that faculty members at their institution rarely conducted SRs. This is in striking contrast to the findings of the Murphy and Boden study, in which none of the human health sciences librarian respondents reported a lack of requests to participate in conducting SRs [9]. Instead, Murphy and Boden noted that the increasing level of demand for human health sciences librarians to participate in SRs was “impacting their capacity to accommodate these requests” and that “accommodation of an increase in requests could be challenging” [9].

There are multiple factors that may contribute to the lack of demand for veterinary librarians to participate in SRs. In particular, it may reflect the fact that SRs and scoping reviews are still relatively uncommon in the veterinary literature [1, 15], which in turn may be due to an insufficient number of good-quality veterinary clinical trials on many topics to enable SRs to be conducted [1, 15–17], a lack of organizations dedicated to sponsoring production of veterinary SRs, and a lack of funding to conduct veterinary SRs [18].

Another key barrier that survey respondents noted was a lack of library polices and guidelines for veterinary librarian participation in SRs, with over two-thirds of respondents indicating that their libraries had no policy or that they were unsure if their libraries had a policy. By contrast, less than one-half of human health sciences librarians surveyed by Murphy and Boden indicated that their libraries had no policy or guideline regarding librarian functions and level of involvement in SRs and scoping reviews [9].

An additional barrier that was not mentioned by any survey respondents is the lack of research-based guidance specific to conducting SR literature searches in veterinary medicine in comparison to human health sciences disciplines [19]. In a comprehensive review of information retrieval methods used for SRs in food animal health and welfare, Wood et al. found that there was relatively little research on search methodology for these disciplines [15].

### Training

A majority of both veterinary and human health sciences librarians rated their training in the traditional librarian SR roles of question formation, database selection, search strategy development, and reference management as pretty good or extensive. Likewise, both groups of librarians reported having less training in nontraditional SR roles. However, veterinary librarians differed from their human health sciences counterparts in that they noted insufficient training as the fourth most significant barrier to participation in SRs, whereas human health sciences librarians noted this as the second greatest barrier to participation in SRs [9].

There is an apparent contradiction between the majority of veterinary librarians rating their training in traditional librarian roles as pretty good or extensive and their indication that insufficient training is a significant barrier to participation in SRs. While the data from this study do not provide a direct answer to this contradiction, a contributing factor might be the paucity of training programs that are targeted to conducting SRs of the veterinary and animal health literature. While there are many well-established training programs available for librarians conducting SRs of the human health sciences literature [20], several leading experts in veterinary SRs have contended that many aspects of search methodology developed for human health sciences SRs should not automatically be applied to veterinary and animal health SRs because of the methodological differences between veterinary and human health sciences research literature [15]. Several specific differences that have been noted were greater use of nonrandomized and observational study designs in veterinary clinical trials and the lack of research evidence on whether publication bias is present in non-RCTs, which are more prevalent in the veterinary literature.

These differences call into question the utility of human medicine RCT search filters and the need for extensive grey literature searches for unpublished clinical trials in veterinary SRs. In light of this question, training focused on conducting veterinary SRs and scoping reviews is needed. In recent years, several workshops on conducting SRs in veterinary medicine and animal health have been offered, but such training opportunities are still relatively rare [21–23].

### Other types of literature reviews

Adherence to a specific search methodology is not typically required in narrative reviews or general literature reviews, such as those done for graduate study theses and dissertations. However, application of relevant SR search methods to these other types of literature reviews that veterinary librarians already participate in could increase the methodological rigor of these reviews as well as raise awareness among veterinary students and researchers of the importance of search methodology to the quality and completeness of literature reviews. For example, SR search methodology employs techniques to ensure that searches are comprehensive rather than selectively aligned with author biases. In SRs, the full line-by-line database search strategies are reported so that readers of the final review can assess for themselves whether the SR data collection process was adequate.

### Limitations

This study has several limitations. First, the small sample size makes findings suggestive rather than definitive. Second, the types of questions in the survey had limited capacity to gather data that could help to explain why certain trends were present among veterinary librarians. Including more short answer survey questions to allow respondents to comment about the questions or conducting interviews with veterinary librarians could have provided a more complete understanding of the reasons for the trends noted in this study. Third, 24 (96%) out of 25 respondents reported some level of participation in SRs in the past. Although the survey did not require respondents to have experience in conducting SRs, librarians without this experience might have chosen not to respond to the survey, so their perspectives might not have been captured in this study. While not extensive, a fourth limitation of this study was the missing data in the survey questions about training and involvement of veterinary librarians in other types of literature reviews. Details of the missing data are noted in supplementary Appendix B in the associated data.

## CONCLUSIONS

The key findings of this study support anecdotal evidence that veterinary librarians participate in SRs to a more limited degree than their human health sciences counterparts. Veterinary librarians experience low demand from veterinary faculty and students to participate in SRs, and veterinary librarians may participate as review team members at significantly lower rates than human health sciences librarians. The main barriers to participation in veterinary SRs that respondents mentioned were lack of library policies, insufficient training, and lack of time.

Participating in SRs is a significant professional opportunity for veterinary librarians to support both veterinary research and clinical practice. Addressing the barriers that veterinary librarians face in participating in SRs will require a sustained, multifaceted approach, particularly in light of the relatively slow uptake of SR methodology by veterinary researchers [15]. Training specific to conducting veterinary SRs and the development of library policies for librarian roles in SRs could facilitate greater participation by veterinary librarians on SR teams and enable librarians to function in a greater range of roles, including coauthorship of reviews.

Further research is needed to address the gaps in veterinary SR search methodology that were identified by Wood et al. as enabling the development of research-based guidelines for information retrieval for veterinary SRs [15]. Additional research is also needed to gain a better understanding of why many veterinary faculty members are not conducting SRs and why they are not asking veterinary librarians to join literature review teams so that policies and research support services in academic libraries can be customized and marketed more effectively to animal health researchers.

Ultimately, demand for veterinary librarians to participate in SRs depends on the development of stronger capacity for knowledge synthesis in the veterinary research community. Although there have been calls in recent years in the research literature to build a “consolidated infrastructure” to fund and provide support for developing animal health knowledge synthesis research, training, standards, resources, and expertise [24, 25], the veterinary infrastructure in these areas remains nascent relative to other health sciences disciplines [18, 19].

If the veterinary research community does not develop a more robust knowledge synthesis research infrastructure, the number of veterinary SRs that are produced will likely remain relatively low for the foreseeable future, as will the demand for veterinary librarian participation in conducting SRs. However, in their current roles as consultants, teachers, and participants in non-SR literature reviews, veterinary librarians can nonetheless play a significant role in increasing awareness among students and faculty of the relationship between search methodology and the quality and completeness of literature reviews and, in so doing, facilitate the uptake of relevant SR search methodology by veterinary researchers.

## SUPPLEMENTAL FILES

Appendix A Questionnaire

Appendix B Associated data

## 

**Lorraine Toews, MLIS**, ltoews@ucalgary.ca, http://orcid.org/0000-0003-4692-0358, Librarian, Veterinary Medicine and Bachelor of Health Sciences, Health Sciences Library, 3330 Hospital Drive Northwest, University of Calgary, Calgary, AB T2N 4N1, Canada, and Adjunct Associate Librarian, Department of Ecosystem and Public Health, Faculty of Veterinary Medicine, 3280 Hospital Drive Northwest, University of Calgary, Calgary, AB T2N 4Z6, Canada
