# Supplementary material for: Benchmarking veterinary librarians’ participation in systematic reviews and scoping reviews
Source: J Med Libr Assoc. 2019 Oct 1;107(4):499–507. doi: 10.5195/jmla.2019.710 (PMC6774563; doi:10.5195/jmla.2019.710)
Supplement: Appendix A [file jmla-107-499-s001.pdf]

JMLA Journal of the Medical Library Association

SC
Supplemental content to
J Med Libr Assoc. Oct;107(4):dx.doi.org/10.5195/jmla.2019.710
www.jmla.mlanet.org
© Toews 2019

Benchmarking veterinary librarians' participation in systematic reviews and scoping reviews

Lorraine Toews, MLIS

APPENDIX A

Questionnaire

Q1. What is your current job title? Select all that apply.

- o Branch head
- o Information specialist
- o Knowledge synthesis librarian
- o Liaison librarian
- o Public services librarian
- o Reference librarian
- o Research librarian
- o Subject librarian
- o Other. Please specify.

Q2. Think specifically about systematic reviews or scoping reviews when answering questions below. Select all that apply.

"A systematic review attempts to collate all empirical evidence that fits pre-specified eligibility criteria in order to answer a specific research question. It uses explicit, systematic methods that are selected with a view to minimizing bias, thus providing more reliable findings."

Higgins JT, Green S, eds. Cochrane handbook for systematic reviews of interventions [Internet]. Version 5.1.0. Cochrane Collaboration; Mar 2011 [cited 2 May 2019]. <http://handbook-5-1.cochrane.org/>.

A scoping review is a type of research synthesis that aims to "map the literature on a particular topic or research area and…to identify key concepts; gaps in the research; and types and sources of evidence to inform practice, policymaking, and research."

Daudt HM, van Mossel C, Scott SJ. Enhancing the scoping study methodology: a large, inter-professional team's experience with Arksey and O'Malley's framework. BMC Med Res Methodol. 2013 Mar 23;13:48.

JMLA Journal of the Medical Library Association

SC
Supplemental content to
J Med Libr Assoc. Oct;107(4):dx.doi.org/10.5195/jmla.2019.710
www.jmla.mlanet.org
© Toews 2019

**Roles**	**Q2a: Have you provided *formal classes or workshops* to your faculty or students on any of the following roles in the past 3 years?** (Excel data file columns D to N)	**Q2b: Have you provided *advice or consultations* to your faculty or students on any of the following roles in the past 3 years?** (Excel data file columns P to Z)	**Q2c: Have you *participated in* any of the following roles on a systematic review or scoping review *team o*n the past 3 years?** (Excel data file columns AB to AL)
Project leader: Write funding grant with team, liaise with funding bodies, chair project meetings, coordinate writing the final report, disseminate review	Yes/No	Yes/No	Yes/No
Project manager: Coordinate, monitor project to ensure review completed within timescale and budget	Yes/No	Yes/No	Yes/No
Literature searcher: Formulate research question	Yes/No	Yes/No	Yes/No
Literature searcher: Select database	Yes/No	Yes/No	Yes/No
Literature searcher: Develop, conduct, report search strategy	Yes/No	Yes/No	Yes/No
Reference manager: Remove duplicate references, track number of references from database searches	Yes/No	Yes/No	Yes/No
Article selector: Select articles based on inclusion/exclusion criteria	Yes/No	Yes/No	Yes/No

Journal of the Medical Library Association

Supplemental content to
J Med Libr Assoc. Oct;107(4):dx.doi.org/10.5195/jmla.2019.710
www.jmla.mlanet.org
© Toews 2019

**Roles**	**Q2a: Have you provided *formal classes or workshops* to your faculty or students on any of the following roles in the past 3 years?** (Excel data file columns D to N)	**Q2b: Have you provided *advice or consultations* to your faculty or students on any of the following roles in the past 3 years?** (Excel data file columns P to Z)	**Q2c: Have you *participated in* any of the following roles on a systematic review or scoping review *team* *o*n the past 3 years?** (Excel data file columns AB to AL)
Data extractor: Extract data from included studies	Yes/No	Yes/No	Yes/No
Article appraiser: Assess the quality of included studies	Yes/No	Yes/No	Yes/No
Data synthesizer: Synthesize results as a narrative or meta-analysis	Yes/No	Yes/No	Yes/No
Report writer: Write up search methods, review report manuscript	Yes/No	Yes/No	Yes/No

JMLA Journal of the Medical Library Association

SC
Supplemental content to
J Med Libr Assoc. Oct;107(4):dx.doi.org/10.5195/jmla.2019.710
www.jmla.mlanet.org
© Toews 2019

Q3. Using the scale below, please rate the training (self-directed or formal) that you have obtained in the context of a systematic review or scoping review. (Excel data file columns AN to AW)

0=None	1=Some	2=Pretty good	3=Extensive

**Role**	**How would you rate your level of training (either self-directed or formal) in each role?**			
Managing projects	0	1	2	3
Formulating the research question	0	1	2	3
Selecting databases and grey literature sources	0	1	2	3
Developing, conducting, reporting the search strategy	0	1	2	3
De-duplicating references, tracking number references	0	1	2	3
Selecting articles for inclusion and exclusion	0	1	2	3
Extracting data from included studies	0	1	2	3
Assessing the quality of included studies	0	1	2	3
Synthesizing results in a narrative or meta-analysis	0	1	2	3
Writing up search methods, reviewing the article manuscript	0	1	2	3

Q4. Which of the following have limited your ability to participate in any role in systematic reviews or scoping reviews? Please select all that apply. (Excel data file column AX)

- o I have received few or no requests to participate in systematic/scoping reviews from veterinary faculty/students at my institution
- o Veterinary faculty/students at my institution rarely conduct systematic/scoping reviews
- o I don't have sufficient training
- o It is not part of my assigned duties
- o I don't have enough time
- o I am not interested
- o It is a low priority service at my institution's library
- o My institution's library does not have the databases needed for systematic/scoping reviews
- o Other. Please clarify.

JMLA Journal of the Medical Library Association

SC

Supplemental content to
J Med Libr Assoc. Oct;107(4):dx.doi.org/10.5195/jmla.2019.710
www.jmla.mlanet.org
© Toews 2019

Q5. Does your library have policies or guidelines to assist you in explaining your potential role(s) and level(s) of involvement to faculty members or students who ask you to participate in systematic reviews or scoping reviews? (Excel data file column AV)

- o Yes
- o No
- o Not sure

Q6. What other types of veterinary literature reviews have you been involved in during the past three years? Please specify. (Excel data file column AZ)

Thank you for your participation in this study.
